# Impact of biobanks on research outcomes in rare diseases: a systematic review

**DOI:** 10.1186/s13023-018-0942-z

**Published:** 2018-11-12

**Authors:** Monique Garcia, Jenny Downs, Alyce Russell, Wei Wang

**Affiliations:** 1 School of Medical and Health Sciences, Edith Cowan University, 270 Joondalup Drive, Joondalup, Perth, WA 6027 Australia; 2 Telethon Kids Institute, The University of Western Australia, Perth, Australia; 3 School of Physiotherapy and Exercise Science, Curtin University, Perth, Australia; 4 Key Municipal Laboratory of Clinical Epidemiology, Capital Medical University, Beijing, China; 5 Taishan Medical University, Taian, China

**Keywords:** Rare disease, Registries, Biobank, Systematic review

## Abstract

**Background:**

Alleviating the burden of rare diseases requires research into new diagnostic and therapeutic strategies. We undertook a systematic review to identify and compare the impact of stand-alone registries, registries with biobanks, and rare disease biobanks on research outcomes in rare diseases.

**Methods:**

A systematic review and meta-aggregation was conducted using the preferred reporting items for systematic reviews and meta-analyses (the PRISMA statement). English language publications were sourced from PubMed, Medline, Scopus, and Web of Science. Original research papers that reported clinical, epidemiological, basic or translational research findings derived from data contained in stand-alone registries, registries with biobanks, and rare disease biobanks were considered. Articles selected for inclusion were assessed using the critical appraisal instruments by JBI-QARI. Each article was read in its entirety and findings were extracted using the online data extraction software from JBI-QARI.

**Results:**

Thirty studies including 28 rare disease resources were included in the review. Of those, 14 registries were not associated to biobank infrastructure, 9 registries were associated with biobank infrastructure, and 6 were rare disease biobank resources. Stand-alone registries had the capacity to uncover the natural history of disease and contributed to evidence-based practice. When annexed to biobank infrastructure, registries could also identify and validate biomarkers, uncover novel genes, elucidate pathogenesis at the Omics level, and develop new therapeutic strategies. Rare disease biobanks in this review had similar capacity for biological investigations, but in addition, had far greater sample numbers and higher quality laboratory techniques for quality assurance processes.

**Discussion:**

We examined the research outcomes of three specific populations: stand-alone registries, registries with biobanks, and stand-alone rare disease biobanks and demonstrated that there are key differences among these resources. These differences are a function of the resources’ design, aims, and objectives, with each resource having a distinctive and important role in contributing to the body of knowledge for rare disease research. Whilst stand-alone registries had the capacity to uncover the natural history of disease, develop best practice, replace clinical trials, and improve patient outcomes, they were limited in their capacity to conduct basic research. The role of basic research in rare disease research is vital; scientists must first understand the pathways of disease before they can develop appropriate interventions. Rare disease biobanks, on the other hand (particularly larger biobanks), had the key infrastructure required to conduct basic research, making novel Omics discoveries, identify and validate biomarkers, uncover novel genes, and develop new therapeutic strategies. However, these stand-alone rare disease biobanks did not collect comprehensive data or impact on clinical observations like a rare disease registry.

Rare disease research is important not only for rare diseases, but also for also common diseases. For example, research of low-density lipoprotein (LDL)-receptors in the rare disease known as familial hypercholesterolemia led to the discovery of statins, a drug therapy that is now used routinely to prevent heart disease.

**Conclusions:**

Rare diseases are still under-researched worldwide. This review made the important observation that registries with biobanks had the function of both stand-alone registries (the capacity to collect comprehensive clinical and epidemiological data) and stand-alone rare disease biobanks (the ability to contribute to Omics research). We found registries with biobanks offer a unique, practical, cost-effective, and impactful solution for rare disease research. Linkage of stand-alone registries to rare disease biobanks will provide the appropriate resources required for the effective translation of basic research into clinical practice. Furthermore, facilitators such as collaboration, engagement, blended recruitment, pro-active marketing, broad consent, and “virtual biobank” online catalogues will, if utilised, add to the success of these resources. These important observations can serve to direct future rare diseases research efforts, ultimately improve patient outcomes and alleviate the significant burden associated with rare disease for clinicians, hospitals, society, and most importantly, the patients and their families.

**Electronic supplementary material:**

The online version of this article (10.1186/s13023-018-0942-z) contains supplementary material, which is available to authorized users.

## Background

Rare diseases (RDs), also known as “orphan” or “neglected” diseases, occur in small proportions of the population. The European Union (EU) consumer-endorsed definition of RDs is those with “life-threatening or chronically debilitating diseases which are of such low prevalence (1 in 2,000 people) that special combined efforts are needed to address them” [1]. Most RDs occur during childhood and are often disabling, incurable, painful, and cause great suffering [2]. In Western Australia (WA), a recent data linkage study found 467 RDs were logged in hospital records with a discharge date between 1 July 1999 and 31 December 2010, accounting for 2% of the WA population [3]. The study also showed approximately 10% of all hospital admissions in WA were related to RDs. Moreover, RDs accounted for 10.5% of total WA hospital expenditure ($395 million) over 1 year [3]. Very few RDs have effective treatments [4], and therefore, RDs continue to place a significant burden on the healthcare system [5].

New diagnostic and therapeutic strategies are urgently needed to manage RDs, with registries being recognised as an effective tool to advance RD research [5]. National and international registries for RDs are necessary to bring together patients to facilitate research, as patient numbers in local jurisdictions for each RD are too few [5]. Orphanet, an online catalogue of over 6000 RDs and directory of expert resources for participating countries, recently stated that RD registries are “the only way to pool data in order to achieve a sufficient sample size for epidemiological and/or clinical research” [6]. For example, the Australian Rett Syndrome Database, established in 1993 to investigate this rare neurodevelopmental disorder, has led to a greater understanding of the natural history of disease, impact of treatment, and facilitated more than 100 research publications on Rett syndrome [7]. As well as clinical data, some RD registries also collect biological samples, such as blood. These samples are processed and stored in specialised freezers as a biobank (BB). BBs, also referred to as “biological specimen banks”, “tissue banks” or “biorepositories”, link a patient’s biological sample to their clinical data, providing detailed phenotypic and genotypic information. The aim of a BB is to then make samples and data available to the scientific community for further studies. The United Kingdom BB is one of the world’s largest, with over 500,000 participants aged between 40 and 69 years [8]. The open-access resource enables investigations of genetic and environmental causes of diseases to improve the prevention, diagnosis, and treatment of diseases affecting the greater community [9]. Whilst BBs require significant commitment, planning and long-term funding, the benefits of drug discovery far outweigh these costs [13]. This is especially so with RDs, where “every sample counts” [14].

RDs have been referred to as “fundamental diseases”, providing opportunities to investigate the “extremes of human pathology” whilst also affording unique insights into normal and abnormal human physiology [10, 11]. This leads to a greater understanding of biological pathways and the identification of therapeutic strategies not only for RDs, but also common diseases [12]. For example, research of low-density lipoprotein (LDL)-receptors in familial hypercholesterolemia, a RD, led to the discovery of statins, a drug therapy that is now used routinely to prevent heart disease [11]. We undertook a systematic review to identify the impact of BBs and interventions derived from BB infrastructure on research outcomes in RDs, and compared research outcomes from stand-alone registries (REG), registries with BBs (REG + BB), and stand-alone Rare Disease Biobanks (RDBB) We also aimed to provide recommendations for practice and policy.

## Methods

### Research design

A systematic review and meta-aggregation was conducted using the preferred reporting items for systematic reviews and meta-analyses (the PRISMA statement) [15]. The Joanna Briggs Institute Qualitative Assessment and Review Instrument (JBI-QARI) method of meta-aggregation was used for critical appraisal of articles, data extraction, and synthesis of data [16]. This qualitative method was developed to mirror the Cochrane’s collaboration processes for quantitative systematic reviews.

### Search strategy

All articles from 1991 to 2016, to include the pre-genomic and genomic era, and published in English were considered. PubMed, Medline, Scopus and Web of Science databases were utilised. The following search terms were used: Rare diseases OR neglected diseases OR orphan diseases AND Biological Specimen Bank OR tissue bank OR registries/standards* OR registries/therapies* OR biobank* OR biorepository.

### Eligibility criteria

Original research papers that reported clinical, epidemiological, basic or translational research findings derived from data contained in a REG, REG + BB, or RDBB were included. For the purpose of this study, a registry was defined as any database that collected any of the following types of data sets: basic, epidemiological, clinical and comprehensive data. All study designs were included. Retrieved articles were initially screened by title and abstract, and if potentially eligible, their full-text was reviewed.

### Critical appraisal

Articles selected for inclusion were assessed using the critical appraisal instruments by JBI-QARI. Two researchers performed the critical appraisal and compared results. In the instance of disagreement, a third party was sought, and consensus was reached.

### Data extraction

Four domains were developed for the data coding sheet – study quality, methodology, type of intervention, and data/specimen collection fields. Each article was read in its entirety and findings were extracted using the online data extraction software from JBI-QARI. Findings were recorded as verbatim quotes of the original articles author’s interpretation of results. An illustration (direct quote) was included to support each finding. Findings were assigned a level of plausibility (unequivocal or credible).

### Data synthesis

Data was synthesised using meta-aggregation analysis [16]. The findings were grouped through similarity of meaning. Categories were developed to describe the concepts within each group of findings, with at least two findings per category. The categories were then grouped into a synthesised finding with at least two categories per synthesis. Categories were further grouped into six themes: basic science, translational science, clinical observation, clinical treatment, study quality, and facilitators and barriers. The synthesised findings constituted the set of recommendations for practice and policy.

## Results

**Fig. 1:**
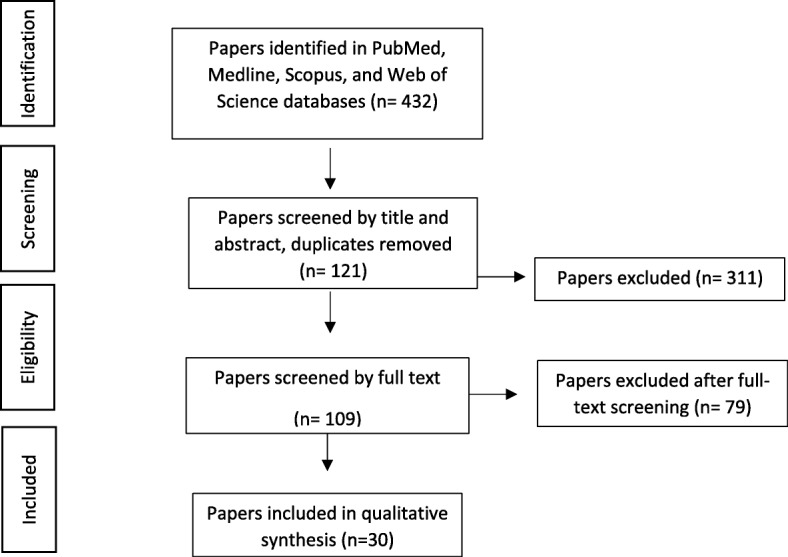
Flow diagram for article section and inclusion of review. The search retrieved 432 citations; 311 were excluded, with full text retrieved for 109. Of those, 79 did not meet the eligibility criteria. A total of 30 articles were included in the review

The initial search retrieved 433 citations. Articles were then screened by title and abstract. Only articles whose abstracts reported results directly pertaining to the primary aims, objectives, and outcomes of the resource itself were included. Articles that reported findings of a study that came about as a consequence of the resource were excluded. As a result, 311 citations were excluded. The remaining 122 articles were retrieved with their full text reviewed. Only articles that reported the following were included: type of resource established, methodology (such as data collection, consent process, recruitment, number of participants, and funding), and primary outcomes of the resource including clinical, epidemiological, basic or translational research findings. Of those, 92 did not meet the eligibility criteria, with a total of 29 articles included in the review Fig. 1.

The 29 studies included 28 RD resources. There were nine REG + BB, 14 REG, and six RDBB. Of the nine REG + BB, six were international networks, and three were national networks. Of the 14 REG, nine were international networks, and five were national networks. Of the six RDBB, two were international networks, three were national networks, and one was a single site initiative. The registries were European (*n* = 12), International (*n* = 7), North American (*n* = 4), Australian and New Zealand (*n* = 3), Japanese (*n* = 1), and Canadian (*n* = 1). Twenty-one registries were established since 2000, with eight established since 2010. Studies were mainly prospective and longitudinal in design, with only a few registries collecting retrospective or cross-sectional data. Disease categories included cancer, genetic, neuromuscular, neurological, lung diseases, cardiovascular, urogenital/renal, autoimmune, autoinflammatory, endocrine, blood, and hereditary ocular diseases.

### Study quality

Registry cohorts ranged from paediatrics to adults or included both children and adults. REG, REG + BB, and RDBB cohort sizes ranged from 23 to greater than 13,500 participants. The total number of biospecimens collected ranged from 46 to over 500,000. The number of research projects emanating from REG, REG + BB, and RDBB ranged from 1 to 784, with the number of research publications ranging from 1 to 255 since the project started.

Twenty registries listed their funding sources: four were funded by the European Commission; three were funded by pharmaceutical companies; two by each of the following including the Department of Health, foundations, institutes, and research trusts; one from either university, charity, society, or benefactor funds; and one from a variety of sources. Four registries reported funding amounts ($170,000 per annum, 1.22 Million, 1.6Million and unrestricted funding). Nineteen registries reported that their data and samples are available to researchers. All registries specified the RD name of interest, yet only five used the World Health Organisation (WHO), International Classification of Disease (ICD) or the Online Mendelian Inheritance in Man (OMIM) coding systems. A list of the registries, and their association with BBs at the time the original article was published, can be found in Table 1.

**Table 1:** List of RD resources

Study ID	Author	Link to resource (reference number)	Resource name	Registry with Biobank (REG + BB)	Stand-alone registry only^a^ (REG)	Stand-alone Rare DiseaseBiobank (RDBB)	Themes associated with resource
N1–001	O’Souji, C	[31]	The Children’s Oncology Rare and Cutaneous NHL registry	2			CO, CT, B
N1–002	Mora, M	[17]	The Eurobiobank Network			2	BS, T, CT, F, B
N1–003	Filocomo, M	[19]	Telethon Network of Genetic Biobanks			2	BS, T, CO, F, B
N1–004	Ebner, K	[27]	The European ARPKD registry	2			CO, CT, F
N1–005	Blain, D	[18]	Eyegene	2			BS, CT, F
N1–006	Bush, A	[32]	European Management Platform for Childhood Interstitial Lung Diseases	2			CT, F
N1–007	Martin, N	[29]	The UK JDM cohort biomarker study and repository Juvenile Dermatomyositis (UK and Ireland) Cohort Biomarker study and Repository for Idiopathic Inflammatory Myopathies	2			CO, CT, F, B
N1–008	Fisher, C	[25]	The PTS registry and biobank network - an AOSpine Knowledge forum tumour study	2			BS, CO, CT, F, B
N1–009	Ugolini, D	[35]	The CREST biorepository			1	F
N1–010	Brandenburg, V	[24]	The German Calciphylaxis registry	1			BS, CO, CT, F, B
N1–011	Struik, M	[48]	The Dutch Lymphangioleiomyomatosis (LAM) registry	1			F
N1–012	Squitieri, F	[49]	Italian Huntington Disease patients - data and tissue bank	1			F
N1–013	Li, J	[13]	Friedrich’s Ataxia fibroblast repository			1	F
N1–014	Zhou, L	[14]	The Tumour Bank at the Children’s Hospital Westmead (TB-CHW)			0	F
N1–015	Bladen, C	[33]	The TREAT-NMD Duchenne Muscular Dystrophy Registries		2^a^		CT, B
N1–016	Webb, S	[30]	The European Registry of Cushing’s Syndrome (ERCUSYN) registry		2		CO, CT, F
N1–017	Sharkey, E	[47]	The NF1 Patient Registry Initiative		2		CO
N1–018	Rodger, S	[23]	The TREAT-NMD care and trial site registry		2		BS, CO, F, B
N1–019	Tilson, H	[26]	The Cryopyrin-associated periodic syndrome (CAPS) registry		2		T, CO, CT, F, B
N1–020	Mistry, P	[28]	The International Collaborative Gaucher Group (ICGG) Gaucher registry		2		CO, CT
N1–021	Evangelista, T	[21]	The UK Facioscapulohumeral muscular dystrophy patient registry		1^a^		BS, CO, CT, F, B
N1–022	Hilbert, J	[22]	The National Registry of Myotonic Dystrophy (MD) and Facioscapulohumeral (FSHD)		1		BS, CO, F
N1–023	Fasnacht, M	[20]	The Swiss Registry for Pulmonary Arterial Hypertension		1		BS, CO, CT, F
N1–024	Downs, J Leonard H, Louise, S	[7, 51]	The Australian Rett Syndrome Database The InterRett Database		1 2		CO, F
N1–025	Korngut, L	[34]	The Canadian Neuromuscular Disease Registry (CNDR)		1		F, B
N1–026	Fehr, S	[50]	The International CDKL5 Disorder Database		2		CO, F
N1–027	Akbarnia, B		The Growing Spine Study Group		2		CO, CT, F
N1–028	Tada, M	[52]	The Rare Disease Bank of Japan: establishment, current status and future challenges			1	F, O

*BS* Basic Science, *T* Translational Science, *CO* Clinical Observation, *CT* Clinical Treatment, *F* Facilitators, *B* Barriers, 0–Single site, 1–National, 2–International

^a^Denotes registries that, in addition to collecting clinical data, also collect genetic information. These registries do not collect or store biological samples, and as such, are still considered registries only

### Themes

The synthesis generated 492 findings, 34 categories, and six themes. The themes were titled basic science, translational science, clinical observation, clinical treatment, study quality, and facilitators and barriers. Synthesised Themes and Categories identified in REG, REG + BB, and RDBB can be found in Table 2.

**Table 2:** Synthesised Themes and Categories identified in stand-alone registries, registries linked to Biobanks, and stand-alone Rare Disease Biobanks

Theme	Theme code	Category	Linked resource (ref.)	Identified in registries linked to Biobanks (REG + BB)	Identified in stand-alone registries (REG)	Identified in stand-alone Rare Disease Biobanks (RDBB)
Basic Science	BS	Omics	[14, 52, 17, 18, 29, 35, 48, 19, 13, 31]	✓		✓
		Biomarker development	[32, 35, 29]	✓		
		Subcohort identification	[13, 25, 35, 21, 27]	✓		✓
		Epidemiology	[48, 33, 21, 24, 26, 20, 22, 29, 35, 23, 30, 19, 34]	✓	✓	✓
Translational science	T	Increased research projects	[14, 33, 21, 23, 17, 30, 19, 22, 29, 18, 34]	✓	✓	✓
		Randomised controlled trials	[48, 33, 34, 32, 22, 23, 21, 27, 29]	✓	✓	
		Biospecimen contribution to studies	[31, 17, 19, 27, 18, 32, 29, 25, 35, 24, 48, 49, 13, 14]	✓		✓
Clinical observation	CO	Diagnosis/survival rate	[28, 29, 25, 20, 19, 50, 51]	✓	✓	
		Natural history of disease	[31, 24, 27, 30, 23, 21, 28, 22, 29]	✓	✓	
Clinical treatment	CT	Diagnostics	[17, 18, 33]	✓	✓	
		Guidelines for treatment	[32, 20, 21, 33, 25, 29]	✓	✓	
		Treatment evaluation	[26, 27, 30, 31, 20, 28, 33, 29, 25, 24]	✓	✓	
Facilitators	F	Benefits to stakeholders	[23, 17, 18, 14, 32, 19, 22, 13]	✓	✓	✓
		Collaborations	[24, 48, 20, 13, 26, 22, 17, 18, 32, 29, 33, 35, 30, 25, 49, 19, 21, 27]	✓	✓	✓
		Engagement	[29, 30, 32, 34, 35]	✓	✓	✓
		Recruitment	[34]	✓		
		Pro-active marketing	[14]			✓
Barriers	B	Challenges	[13, 17, 19, 21, 23–26, 29, 31, 33, 34]	✓	✓	✓

#### Basic science

##### Omics

Both REG + BB and RDBB impacted on RD research outcomes by facilitating Omics studies and discoveries. No evidence of basic science research being conducted in REG was found. The basic science discoveries included the characterisation of new syndromes, biomarker discovery and validation, elucidation of biological pathways involved in disease, molecular modelling of pathogenic variants, characterisation of epigenetic factors involved in disease expression, genotype-phenotype correlations, molecular analysis of DNA methylation, chromatin structure, gene-transfection and gene-silencing studies, studies involving growth factors and cytokines, identification of new gene and novel mutations, and exon-skipping [17–19].

##### Epidemiology and studies of phenotype

REG, REG + BB, and RDBBs all impacted on RD research outcomes with regards to epidemiological studies including the incidence/prevalence of the disorder and survival, natural history, relationships between genotype and phenotype, and understanding the burden of disease. Captured epidemiological data in this review included age, characterisation of symptoms, gender distribution, ethnic background, provision of care at different sites, diagnosis of patient, and data pertaining specifically to the disease of interest [20–25].

#### Translational science

##### Availability of biospecimens for research

This review found that both REG + BB and RDBBs impacted on RD research outcomes in translational science by contributing biological specimens to research projects, leading to new therapies to treat RD. REG could not contribute biological specimens, and so lacked the capacity to contribute to the development of new diagnostic tools and therapies. Both REG + BB and RDBB in this review donated biological samples to pharmaceutical companies (e.g.Pfizer), consortiums, and international studies [19].

##### Clinical trials

This review found that REG, REG + BB, and RDBB all impacted on RD research outcomes in clinical trials with regards to increased patient recruitment and novel safety monitoring approaches. It was found that REG may offer the possibility of study designs other than randomised controlled trials (RCT), hence they are often more advantageous [26]. Unlike RCTs, registries may have access to a large cohort, and have no dictated treatment regimens or strict inclusion criteria [26]. REG have the capacity to collect information from patients in a real world setting during routine clinical care, and because they are observational, all patients receiving treatment can be included, irrespective of dosage. This brings sound external validity as ‘registry enrolled patients’ generally have an increased baseline risk than ‘RCT enrolled patients’. Further, the research period of observation by registries is longer than most RCTs, allowing long-term follow up of new approved therapies.

##### Increased research activity

**Fig. 2:**
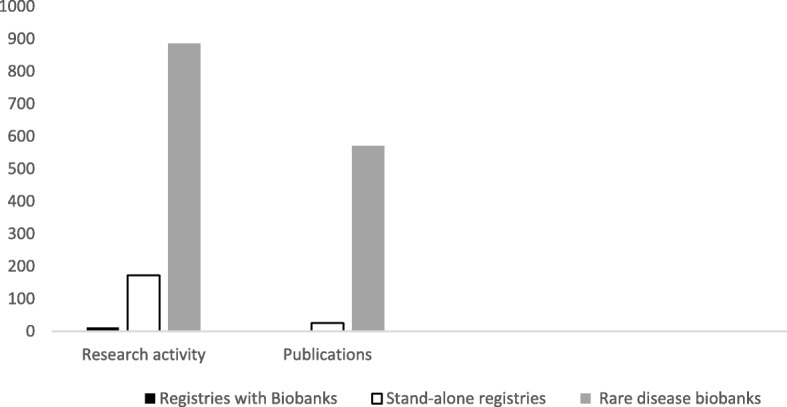
Research impact of RD resources. In this review, research projects and enquiries totalled 886 for RDBB, 172 for REG, and 12 for REG + BB. Further, research publications totalled 571 for RDBB, 26 for REG, and 0 for REG + BB

There is overwhelming evidence that REG and REG + BB significantly increase the number of research projects, and this is amplified when it is a member of a network. Comparatively, RDBBs generated quantifiably more research activity and publications than REG and REG + BB. For example, the use of samples from a European BB network has been acknowledged in 255 publications from 2004 to 2013 [17]. An Italian network provided thousands of samples to national and international researchers over a 5-year period [19]. This led to 784 research projects, with over 250 scientific publications from 2008 to 2012 [19]. In this review, research projects and enquiries totalled 886 for RDBB, 172 for REG, and 12 for REG + BB. Further, research publications totalled 571 for RDBB, 26 for REG, and 0 for REG + BB. This low number from REG + BB may be attributed to the fact that the majority of these resources had only recently been established (Fig. 2).

#### Clinical observation

##### Natural history of disease

Both REG and REG + BB gained insights into the natural course of disease. No RDBB in this review contributed to uncovering the natural history of disease due to the lack of phenotypic data. Registries in this review observed factors that accelerated or slowed development of disease, understood better the resultant disease sequelae, and made new findings regarding disease progression [20–30].

##### Diagnosis, survival rates, patient outcomes

REG, REG + BB, and RDBBs all impacted clinical observation outcomes in regard to diagnosis, survival rates and patient outcomes. REG in this review observed long delays between symptom onset and diagnosis, with multiple consults by specialists observed prior to gaining a confirmed diagnosis [7, 20, 29, 31]. RDBBs had the capacity to store samples for clinicians from undiagnosed patients with the view at future diagnosis, providing retrospective diagnoses [19]. Further, it was found that REG could establish survival rates for various RDs, as well as outcomes at follow-ups in this review [28].

#### Clinical treatment

##### Diagnostics

This review found that both REG + BB and RDBBs supported the development of new diagnostic testing methodologies, whilst REG contributed to observing which participating centres lacked appropriate diagnostic criteria. [17, 18, 23]

##### Guidelines for treatment

Both REG and REG + BB impacted on clinical treatment. RDs commonly lack evidence-based treatment protocols, attributed to the low number of patients seen at any one centre. REG in this review facilitated multi-centre collaboration, which in turn led to discussions among experts regarding treatment protocols and best practice [20, 21, 25, 29, 32, 33]. This contributed to the management of disease, impacting on patient outcomes.

##### Treatment evaluation

REG and REG + BB impacted on treatment evaluation for RD. In this review, no RDBB was directly involved in treatment evaluation studies. Existing therapies and surgical interventions, and their outcomes, were observed. This led to a greater understanding of which therapies affected disease course [20, 24–31, 33].

#### Facilitators

##### Benefits to stakeholders

REG, REG + BB, and RDBB all benefit stakeholders. This review found that participants, patient advocacy groups, researchers, and clinicians all benefited from participation in RD resources [13, 14, 17–19, 21–23, 32].

##### Collaborations

This review found collaborations between all three RD resources and various stakeholders are vital to the success of the registries aims and objectives. All resources collaborated with numerous groups including hospital sites, academic centres, clinicians, patients, scientists, patient advocacy groups, pharmaceutical and diagnostic industries, societies, foundations and other registries. Collaboration was local, regional, national, or international. Collaborative approaches facilitated review and discussion of treatment protocols, improving treatment outcomes [13, 14, 17–22, 24–27]. Continuous engagement assisted clinicians with follow-up, with more complete data being reported. International collaborations increased patient cohort size, leading to increased interest from industry.

##### Engagement

Engagement strategies reported by all resources included the international nature of the registry, ongoing communication between the registry and participating sites, collaboration, methods of recruitment, using data collection forms in place of clinical notes to ease the burden of form filling, inclusion of any interested clinics to increase participant numbers, and equal sharing of funding leading to continuation of data collection even when the funding ceased [29, 30, 32, 34, 35].

##### Recruitment

All resources in this review employed novel methods for the recruitment of RD patients. This lateral thinking is especially important given the small number of patients that are scattered geographically [34].

##### Pro-active marketing

A strategic, targeted, pro-active marketing approach demonstrated how even a single BB site can have a significant impact on RD research outcomes, and can contribute to key research studies throughout the world [14].

#### Barriers

Challenges are reported by all three resource types. Challenges faced by REG included incomplete data sets, data accuracy (error), lack of follow-up data, lack of standardisation, and funding restrictions [17, 19, 21, 23–26, 29, 31, 33, 34]. Another challenge was the ability to reach, recruit and capture all patient cases. Challenges faced by REG + BB and RDBBs in this review included difficulties when implementing next-generation sequencing (NGS) due to legal and ethical concerns [19]. Current informed consent can be restricted to that patient’s particular disease, lacking the necessary broad consent to implement NGS [19]. Moreover, governing the sheer volume of information generated by NGS required additional considerations such as when managing “incidental findings” [19]. It was also found that BBs which cover a broad range of diseases are limited in their ability to reach a critical mass for a particular disease category [13].

The aforementioned challenges are just some of the barriers faced by resources in the process of sample acquisition and storage. Governance Frameworks need to be developed by resources to overcome these obstacles. Such an example is the framework that has been developed, the Office of Population Health Genomics, the Western Australia Health Department [53]. The framework, titled “Guidelines for human biobanks, genetic research databases and associated data” provides a comprehensive manual for registries and biobanks and considers important constraints arising from the use of biological specimens [53]. Issues such as establishment of the resource, governance, ethics, participation (including enrolment, consent/assent, and withdrawal processes), protection of samples and data (privacy, confidentiality, security), standard operating procedures, access of samples (access regulations, return of incidental findings), and benefit sharing (intellectual property, income generation, royalties) are discussed [53]. Rare Disease sample collection, however, is fraught with even more unique barriers, as considered by Tada et al., who reports difficulties in bank operation, updating sample information, and technical improvement [52]. The Telethon Network of Genetic Biobanks documents insightful solutions in overcoming these challenges, specifically for RD. [19] Their report details governance, management and IT framework solutions to achieve standardization and best practice, whilst also considering sustainability, regulations and national/international laws [19].

## Discussion

This review sought to identify and compare the impact of REG, REG + BB, and RDBB on research outcomes in RD. We analysed and compared the research endpoints of three specific resource populations: REG, REG + BB, and RDBBs. Findings were grouped into themes: basic science, translational science, clinical observation, clinical treatment, facilitators, and barriers. We observed key differences among the research endpoint variables between all three RD resources. Most notably, REG + BB and RDBBs included basic science (Omics) as a research endpoint, observed to lie exclusively within the domain of REG + BB and RDBBs. In comparison to REG, the inclusion of basic science as a research endpoint variable in REG + BB and RDBBs was found to have significant and far-reaching consequences by way of facilitating translational research, leading to the discovery and development of new treatments and therapies for RDs. Importantly, REG of RD are often the only resource for the disease of interest [36]. REG are gradually being recognised as a global priority in RD research; the essential “building blocks” for RD epidemiological, clinical research and post-marketing studies [37]. We found REG led to a greater understanding of the natural history of disease, established consensus-driven treatment protocols, replaced clinical trials, and ultimately improved patient outcomes. Despite these benefits, REG were restricted in their smaller capacity to contribute to basic research, attributable to a lack of infrastructure required to conduct the necessary laboratory-based investigations.

Both REG + BB and RDBBs contributed to basic research. Findings included novel Omics discoveries, biomarker development (screening, validation, replication and clinical trial), gene identification, elucidation of biological and cellular pathways, models for drug-screening, and therapeutic discoveries. Further, basic research studies are made possible through the availability of human biological specimens [14]. It is only through the collection and investigation of human biological samples matched to clinical data, such is the case with REG + BB and RDBBs, that novel diagnostic, prognostic, and therapeutic avenues can be developed [25]. This is particularly important considering drug innovation for RD has, in recent years, become progressively focused on Omics studies, with the identification of molecular targets leading to the development of new therapies [10]. The development of new therapies for RDs is critically significant as they can be of a life-saving nature. This is best demonstrated by Strimvelis, the first ex vivo stem-cell gene-therapy for children to gain marketing approval anywhere in the world [38]. In spite of the ground-breaking success of Strimvelis, Fondazione Telethon remains committed to its original focus of basic research, stating “basic science is the foundation on which future treatments will be developed” [12]. This provides a powerful message to RD stakeholders; scientists must first understand the pathways of disease before they can develop appropriate interventions.

The premise that basic science is the key component in REG + BB and RDBBs for the discovery and development of new therapies is consistent with statements from the Eurordis position paper on research priorities for RDs 2014–2020, a recent joint declaration by the European Organisation for Rare Diseases (EURORDIS), the National Organization for Rare Disorders (NORD) and the Canadian Organization for Rare Disorders (CORD) 10 key principles for RD patient REG, and the *WA Rare Diseases Strategic Framework 2015–2018* [5, 37, 39]. In addition to these findings, we found several factors which, when utilised, served to strengthen the success of RD resources. Collaboration, engagement, blended recruitment, pro-active marketing, broad consent, and “virtual BB” online catalogues were all unique facilitators which enhanced the success of REG, REG + BB, and RDBBs [14, 19, 34].

Despite their similarities in contributing to Omics research, differences do exist between REG + BB and RDBBs. The number of samples stored in REG + BB compared to RDBBs was found to be entirely a function of the resources, aims and objectives. REG + BB most often stored small sample numbers, with most resources storing only several hundred samples (such as The German Calciphylaxis registry, which reported storing 253 samples). This was not always the case, however, with the Eyegene registry collecting 4400 rare genetic eye disorder samples. When compared to REG + BB, the number of samples stored in RDBBs were typically much larger, with samples stored being in the thousands. For example, the Telethon Network of Genetic Biobanks reported storing 75,900 samples from patients with RDs. There were, however, small RDBBs that had collected only 50 samples (such as the Friedrich’s Ataxia fibroblast repository). Li et al. reported that smaller RDBBs have their advantages over larger RDBB networks in the sense that they can focus on a single diseases or syndromes, or group of diseases, and can successfully accumulate significant numbers of cell lines, whilst developing an intimate understanding of the disease [13].

REG + BB and RDBBs both have the capacity to collect, store and conduct basic science investigations. It was found RDBBs reported high quality control measures for their samples. For example, in addition to standard sample collection, the Japanese RDBB performs human leukocyte antigen (HLA) analysis and mycoplasma (MC) testing to their acquired samples. The HLA complex plays a key role in the immune system response. Therefore, the Japanese RDBB provided scientists not only with biological samples, but also with highly specialised HLA background data with every specimen. Further, MC are known to infect cell samples, which can lead to part of the cells metabolism being affected. This RDBB reported an MC contamination rate of 22.4% in their 1500 cell samples, and subsequently introduced the MC test to ensure high-quality sample management [52].

Key differences between REG, REG + BB, and RDBBs, were observed in with regards to the number of diseases each resource focused on. Both REG and REG + BB were disease-specific resources, and as such focused their research efforts on either a single disease in a specific population (such as the Swiss registry for pulmonary arterial hypertension in a paediatric population) [20], or a group of diseases (such as The UK JDM cohort biomarker study and repository Juvenile Dermatomyositis (UK and Ireland) Cohort Biomarker study and Repository for Idiopathic Inflammatory Myopathies) [29]. This was not usually the case for RDBBs in this review, which usually focused on multi-disease sample collection. For example, the RDBB of Japan collected samples from 102 different RDs [52], and the Telethon Network of Genetic BB collected samples from over 750 different RDs [19].

Another significant finding from our analyses is the difference in data collection methodologies between REG, REG + BB when compared to larger RDBBs. We found both REG and REG + BB typically collected comprehensive clinical and epidemiological data. For example, The UK Facioscapulohumeral muscular dystrophy patient registry collected not only all the items in the minimal dataset as identified at the 171st ENMC (European Neuromuscular Centre) workshop, capturing demographics, genetics, motor function, and age at onset of disease, but also collected highly encouraged data such as eye, hearing, respiratory status and family history [21]. Further, through validated questionnaires, the registry collected outcomes relating to pain, quality of life, and scapular fixation. Similar to REG and REG + BB, comprehensive data collection was also a feature of small RDBBs. Ugolini et al. stated that small RDBBs with a low number of specimens should be compensated by the high quality of linked clinical and epidemiological data [35]. Limited resources can be overcome by the biorepository through specialisation [13, 35]. Larger RDBBs, however, most often collected minimum data sets. For example, the Telethon Network of Genetic Biobanks collected patient particulars (name, date of birth, etc.), phenotype (affected/not affected), anamnestic data (presence of consanguinity etc.), diagnosis data (modality, centre performing diagnosis), and sample data (code, type, data of collection, etc.) [19].

Both REG and REG + BB contributed to clinical observation and clinical treatment, but this was beyond the scope of the RDBBs function in this review.

We demonstrated that there are key differences among REG, REG + BB, and RDBBs. These differences are a function of the resources’ design, aims, and objectives, with each resource having a distinctive and important role in contributing to the body of knowledge for RD research. That said, this review underscores the unique scope and utility of REG + BB, which has the function of both REG (the capacity to collect comprehensive clinical and epidemiological data) and RDBB (the ability to contribute to Omics research). Linkage of BBs to established RD registries offers a practical, cost-effective, and impactful solution for RD research. This is an important observation that can serve to direct future RD research efforts.

### Strengths and limitations

A limitation of this study is that our chosen search terms used to locate all necessary and relevant RD registries and associated BBs may have led to a “filed-effect” of articles, precluding other valuable studies from our review by default. In addition, there is potential that additional databases have been newly established due to the study of RDs growing in momentum.

## Conclusion

RDs are still under-researched worldwide. We examined the research outcomes of three specific populations: REG, REG + BB, and RDBBs. Whilst REG had the capacity to uncover the natural history of disease, develop best practice, act as “real-world” studies supporting clinical trials, and improve patient outcomes, they were limited in their capacity to conduct basic research. The role of basic research in RD research is vital; scientists must first understand the pathways of disease before they can develop appropriate interventions. RDBBs, on the other hand (particularly larger RDBBs), had the key infrastructure required to conduct basic research, making novel Omics discoveries, identify and validate biomarkers, uncover novel genes, and develop new therapeutic strategies. However, RDBB did not collect comprehensive data or impact on clinical observations like REG. This review made the important observation that REG + BB had the function of both REG (the capacity to collect comprehensive clinical and epidemiological data) and RDBB (the ability to contribute to Omics research). We found REG + BB offers a unique, practical, cost-effective, and impactful solution for RD research. Linkage of REG to RDBBs will likely provide the stronger foundation of resources required for the effective translation of basic research into clinical practice. This is an important observation that can serve to direct future RD research efforts. Furthermore, facilitators such as collaboration, engagement, blended recruitment, pro-active marketing, broad consent, and “virtual BB” online catalogues will, if utilised, add to the success of REG, REG + BB, and RDBBs [14, 19, 34].

### Recommendations

The following evidence-based recommendations are derived from this systematic review and align with the WA Rare Disease Strategic Framework 2015–2018, and the joint declaration of 10 key principles for RD patient registries by the European Organisation for Rare Diseases (EURORDIS), the National Organization for Rare Disorders (NORD) and the Canadian Organization for Rare Disorders (CORD).

1. Established stand-alone registries (REG) should be identified, and, where appropriate, consider extending their research scope to include omics investigations as a research endpoin [20].
2. Established stand-alone registries (REG) choosing to extend their research scope to include omics investigations should collaborate with stand-alone rare disease Biobanks (RDBBs) for the collection, processing, and storage of biological samples, that can then be matched to clinical data in the registry [24].
3. The collaboration of REG with RDBBs should enable a cross-over of each respective resources functions (REG – comprehensive clinical data; RDBB – omics investigations) to now have the unique function of a REG + BB.
4. REG, REG + BB, and RDBBs resources should all consider utilising the following facilitators to enhance the success of the resource; engagement, pro-active marketing, and achievement of informed consent based on broad research goals.
5. REG + BB and RDBBs should consider establishing an online “virtual BB” online catalogue to make their resource known, and should create a steering group consisting of representatives from the following stakeholders; patients, patient support groups, clinicians, and researchers to guide research directions and activities [19].
6. REG, REG + BB, and RDBBs should adopt a “blended recruitment” approach, ensuring the largest possible geographical reach, with direct (patient) or indirect (clinician) enrolment [34].

## Additional file

Additional file 1: Case examples. [40–46]. (DOCX 78 kb)

## Abbreviations

ADA-SCID: Severe combined immunodeficiency due to adenosine deaminase deficiency
BB/s: Biobank/s
CAPS: The Cryopyrin-associated periodic syndrome registry
CNDR: Canadian Neuromuscular disease registry
CORD: Canadian Organization for Rare Disorders
CREST: Cancer of Respiratory Tract Biorepository
CT: Computed topography
DMD: Duchene muscular dystrophy
DNA: Deoxyribonucleic acid
EBB: EuroBioBank
EURORDIS: European Organisation for Rare Diseases
FT: Fondazione Telethon
ICD: International classification of diseases
JBI: Joanna Briggs Institute
JBI-QARI: Joanna Briggs Institute Qualitative Assessment and Review Instrument
MiRNA: MicroRNA
NGS: Next-generation sequencing
NORD: National Organization for Rare Disorders
NSCLC: Non-small cell lung cancer
OMIM: Online Mendelian Inheritance in Man
PAH: Pulmonary arterial hypertension
qPCR: Quantitative polymerase chain reaction
RCT: Randomised controlled trials
RD/s: Rare disease/s
RNA: Ribonucleic acid
TB-CHW: Tumour Bank at the Children’s Hospital Westmead
TNGB: Telethon Network of Genetic Biobanks
TREAT-NMD: Network for neuromuscular diseases
UK: United Kingdom
USA: United States of America
WHO: World Health Organisation
