# Supplementary material for: Impact of biobanks on research outcomes in rare diseases: a systematic review
Source: Orphanet J Rare Dis. 2018 Nov 12;13:202. doi: 10.1186/s13023-018-0942-z (PMC6233271; doi:10.1186/s13023-018-0942-z)
Supplement: Supplementary file 1 — Case examples. [40–46]. (DOCX 78 kb) [file 13023_2018_942_MOESM1_ESM.docx]

**Case Example –Omics discoveries^35, 40^**

In 2011, utilising samples entirely from the Cancer of Respiratory Tract (CREST) biorepository that was primarily established to study mesothelioma (a rare and serious form of lung cancer), a study was conducted to evaluate new biomarkers for non-small cell lung cancer (NSCLC), the most common form of lung cancer.^35, 40^A large proportion of lung cancers (>75%) are detected in the later stages of the disease, attributable to a lack of appropriate screening tools for large numbers of people. Whilst computed topography (CT) screening has been used for at-risk individuals, it is not ideal. For example, approximately 50% of tumours detected by CT are benign, and for every death prevented using this method, two invasive procedures resulting from false-positive results will occur. Late detection using current methods has resulted in poor prognoses for lung cancer patients. Conversely, early detection leads to improved patient outcomes. Therefore, the rationale for biomarker development in lung cancer was for earlier detection of NSCLC, which may lead to improved survival rates. The study aimed to identify serum-based biomarkers for NSCLC. Blood-serum was obtained from 22 participants (11 with early-stage NSCLC and 11 controls) provided by the CREST biorepository. Using microRNA (miRNA) profiling on total RNA, the study found the expression of two miRNAs (has-miR-1254 and has-miR-574-5p) to be significantly elevated in NSCLC cases when compared to controls. Quantitative polymerase chain reaction (qPCR) validated the results, with the authors concluding the findings justified additional consideration and validation of these serum-based biomarkers for early-stage non-small cell lung cancer. The CREST biorepository demonstrates how RD BBs can impact on outcomes (biomarker development) in basic science, not only for RDs (mesothelioma), but also common disease (NSCLC). Moreover, the CREST biorepository stores blood samples over many years, and includes banked samples from individuals prior to their lung cancer diagnosis. This makes RD BBs invaluable for testing the applicability of NSCLC serum-based biomarkers, an opportunity not afforded to other biomarker studies.

**Case Example –Epidemiology and Studies of Phenotype^33, 41, 42, 43, 44^**

TREAT-NMD, a registry network for neuromuscular diseases, provides evidence of how RD initiatives can impact on genetic epidemiological outcomes. Duchenne Muscular Dystrophy (DMD) is a progressive neuromuscular disease caused by mutations in the DMD gene, leading to a depletion of dystrophin within the muscle.^41^ The TREAT-NMD DMD global database is thought to be the world’s largest cohort of DMD mutations, containing over 7,000 mutations.^33, 42^ The initiative collects and compares information regarding DMD mutations (such as location of mutation). Gaining an insight into the type and frequency of mutations that cause DMD is invaluable for genetic diagnosis, basic science, clinical care, and personalised/targeted therapy.^33^

Analysis of the TREAT-NMD DMD Global database showed that, regardless of geographical location, the most prevalent mutation are large deletions (68%) with exon 45 being the most common deletion (reported 316 times). Of the 7,149 mutations, 5,684 (80%) are insertion/deletion (INDELS) mutations, and 1,445 (20%) are point mutations. Half of all point mutations (10% of total mutations) are nonsense mutations: point mutations that lead to a premature ‘stop’ codon being introduced into the amino acid sequence. This usually results in a non-functional protein, causing disease. A potential DMD therapy that has achieved marketing approval is nonsense stop codon read-through therapy.^43^ This treatment selectively induces ribosomal read-through of premature stop codons but not normal stop codons. The database identified 317 mutations (4%) with a premature TGA stop codon, 215 (3%) with a TAG stop codon, and 194 (3%) with a TAA stop codon, that would potentially benefit from this available therapy. Another therapy for DMD is exon-skipping technology.^44^ Exon-skipping takes advantage of the fact that internally deleted dystrophins can be partly functional.^44^ Mutations were identified in the database that would potentially benefit from exon-skipping, such as skipping of exon 51 (14% of all mutations) and 53 (10%). This example highlights how registries can impact on epidemiological outcomes and can assist in identifying patients who may benefit from already existing therapies.

**Case Example – Clinical Trials^26^**

The Cryopyrin-associated periodic syndrome (CAPS) registry is an example of a registry being used in place of a RCT for post-marketing purposes.^26^ CAPS are a group of rare, hereditary autoinflammatory diseases. Symptoms, such as fever and systemic inflammation, present as recurrent episodes throughout the entire lifetime of the patient, and can become life-threatening. Canakinumab, a monoclonal IL-1-β antibody, is an approved treatment for CAPS patients. Like all RDs, the clinical development of Canakinumab recruited a very limited number of patients; therefore, post-approval monitoring to assess the short and long-term safety and efficacy was critical. The CAPS registry was established in 2009 as an online, observational registry with the aim of gathering information regarding the natural history of disease as well as the beneficial and adverse effects of treatment over a 5-year period. With no exclusion criteria, protocol-mandated visits, or procedures, physicians successfully collected data from 241 CAPS patients over 5 years during routine visits to clinic, significantly more than the original drug approved dossier of 78 CAPS patients over a 3.5-year period. An update of the safety profile of Canakinumab in 2013-2014 reported no new or unexpected safety concerns, with no loss of efficacy of the drug.^47^ Moreover, the findings were consistent with the previous clinical trials.

**Case Example – Diagnosis, Survival Rates, Patient Outcomes^7, 48^**

Established in 1993, the Australian Rett Syndrome Database collects data pertaining to, among other factors such as understanding the natural history of disease, challenges associated with obtaining a diagnosis. ^7, 48^ In particular, the database facilitated studies which found that a limited awareness of Rett Syndrome by clinicians led to difficulty in diagnosing the condition.^7^ This was attributed to a high degree of variability in the clinical presentation of Rett Syndrome. As a result, children were either misdiagnosed (for example, with autism or global developmental delay), or not diagnosed at all. The registry also found that, despite the availability of genetic testing for confirmation of a clinical diagnosis of Rett Syndrome via the MECP2 mutation, the test itself was not considered, as some patients did not present with the “classic” symptoms of Rett syndrome (such as high levels of regression). The database found the need for clinicians to be aware of the complex and highly variable presentation associated with Rett Syndrome, so that families were able to make clear and informed choices for their child after a diagnosis had been obtained.

**Case Example – Natural History of Disease^24^**

A novel finding regarding the natural history of disease was uncovered through the German Calciphylaxis registry.^24^ Calciphylaxis is a very RD that carries a high mortality. It is frequently found in patients with end-stage renal disease on dialysis, and manifests as severe skin ulcerations and calcification of cutaneous arterioles.^24^ The German registry was established to identify potential risk factors for disease, clinical practice methods, and biomarker analysis. This was achieved by collecting clinical information as well as blood samples, stored in the registry’s BB. Whilst the registry validated previous reports that end-stage renal disease appears to predispose patients to Calciphylaxis, the novel finding came from the laboratory testing on patient samples, which measured serum calcium, phosphorus and parathyroid hormone levels. The biochemistry results found that parathyroid hormone levels in Calciphylaxis patients were unexpectedly low. The authors recommended that future studies need to explore the trend and time course of these biochemistry markers (calcium, phosphorus, and parathyroid hormone) in the months leading up to Calciphylaxis development.

**Case Example – Diagnostics^17^**

The EuroBioBank (EBB), the first BB network in Europe (and also a partner of the TNGB) collects, processes and stores biological samples (such as DNA and tissue) for provision to the RD scientific community.^17^ One partner of the EBB, known as the Instituto Nazionale Neurologico Carlo Besta, provides samples for new diagnostic tests when they become available. This provides another utility of a BB by way of supporting diagnostic development.

**Case Example - Guidelines for Treatment^29^**

The Juvenile Myositis registry is one such example of how registries can impact on treatment guidelines.^29^ Juvenile Myositis is a group of rare, chronic inflammatory disorders in childhood, affecting muscles and other organs. This disorder is associated with a high level of morbidity and mortality. There is a severe lack of evidence-based treatments for Juvenile Myositis, with almost no level-1 evidence from trials regarding therapies, and little is known about the underlying mechanisms of the disease. Through collaboration with multiple centres, the registry facilitated discussion among experts regarding current treatments. It was observed that early management of Juvenile Myositis improved treatment outcomes, whereas a delay in treatment led to poorer outcomes. The outcome was a shift in practice; specifically, widespread adoption of earlier and more aggressive treatments. In addition, the assessment process for children with Juvenile Myositis changed in participating centres as a result of the registry. The registry employed the Childhood Myositis Assessment Scale, a tool previously validated to assess muscle function, as part of their data collection process. As a result, this assessment tool has since gained widespread use throughout the UK for management of Juvenile Myositis, and is now part of routine clinical practice in participating centres. The registry is currently working on establishing treatment protocols for this disease.

**Case Example – Treatment Evaluation^20^**

The Swiss Registry for Pulmonary Arterial Hypertension (PAH) collects information pertaining to paediatric PAH, a rare condition leading to high blood pressure in the lungs of affected children.^20^ PAH has a poor prognosis if left untreated. Information regarding treatment outcomes for paediatric PAH are scarce. The Swiss registry evaluated treatment outcomes from 23 paediatric PAH patients, with a median follow-up of 3.47 years. Therapies included Bosentan, Sildenafil, and inhaled Iloprost, administered in isolation or in combination. Whilst the treatments were heterogeneous, it was found that the majority of PAH patients achieved stabilisation of their condition under these current available therapies. Further, some patients demonstrated an increased exercise tolerance with improved functional status on these therapies. The authors highlighted the usefulness of such a registry for gathering vital information on therapies for RD.

**Case Example – Benefits to Stakeholders^18^**

Eyegene, a registry and BB dedicated to phenotyping and genotyping rare inherited eye diseases, provides a comprehensive example of how registries benefit numerous stakeholders.^18^. Established by the National Eye Institute, Eyegene’s reach encompasses the USA and Canada. Patients diagnosed with genetic eye diseases are clinically characterised, and have their DNA stored in a BB. Research is then conducted into the underlying pathogenesis of disease. Patients participating in the initiative benefit by being involved in current research which may elucidate the genetic cause of their condition. Eyegene found that patients chose to participate in research studies even when they knew the genetic cause of their disease. Patient support groups presented clinically characterised, previously genotyped individuals to Eyegene for participation, benefiting by expanding their access to research. Clinicians referring their patients to Eyegene benefited by receiving a molecular diagnosis of their patient, confirming the initial clinical diagnosis. Eyegene has provided diagnostic results to over 55% of participants enrolled, and has over 4,400 samples stored with Eyegene. This may help to better monitor and manage respective inherited eye conditions. Furthermore, through Eyegene, both clinicians and patients gain access to information regarding the availability of clinical trials. Finally, the scientific community benefited from Eyegene by accessing clinical data linked to biological samples, which progressed research in the field of inherited eye diseases. For example, Eyegene initially tested 20 genes over 9 disease categories, but now tests more than 100 genes over 35 categories, the result of gene discovery through the network.

**Case Example – Collaborations^38^**

The approval of Strimvelis required extensive collaboration between numerous stakeholders, establishing a new paradigm for RD research. Cooperation was facilitated between academia, not-for-profit organisations, and the pharmaceutical/biotech industry, evidencing how this transparent collaboration overcame the numerous barriers associated with drug development for RDs.^38^  The combined efforts that accelerated the commercialisation of Strimvelis resulted in a “turning point for the field” and has charted a “clear path” for similar gene therapy developments.^38^ The success of the methods developed for the success of Strimvelis extends beyond the obvious benefits to the patient, with the strategies employed for quality assessment, manufacturing, administration of the drug in the clinical setting, and policies for drug cost and reimbursement serving as a precedent for future efforts.^38^

**Case Example – Engagement^19^**

The TNGB attributed 4 major strengths to the success of their national network.^19^ Firstly, the Coordination Office, managed by the Coordinator of the entire network, ensured harmonisation and standardisation of all operating procedures (including collection, processing, and storage of samples and data) throughout the BB. The TNGB reported that maintaining interoperability throughout the BB has prevented siloing and disorganisation from individual efforts. Secondly, a unique collaborative model between the TNGB and various patient support groups for RD in Italy was established. Patient support groups have always had representation on the advisory board since the inception of the network, providing insight and feedback on governance issues such as ethics, consent, and confidentiality. In addition to this role, a “coordinator emeritus” was appointed to support the coordinator by the TNGB to liaise between the patient support groups in Uniamo, an Italian federation of over 100 RD associations. The coordinator emeritus initiated meetings and workshops with the aim of fostering trust and interest among patients and their families regarding the concept of the BB, and how it can provide a resource for future RD research. The TNGB reported that there is a significant increase of interest in the BB, and that patient and family involvement have been vital for both reaching a critical mass of biological samples, as well as taking the patients’ needs and concerns into account. Thirdly, the TNGB used a novel approach in dedicating a specific patient support group to one of the BB in the network. Termed a “framework agreement” and there are now six such agreements in place. Within the framework, patient groups can promote and cofound research projects with the BB they are partnered with, with two projects now developed as a result of this agreement. Lastly, the TNGB attributes another strength to its success through its online catalogue. The “virtual biobank” lists all samples stored within the BB network online, making some 75,900 samples visible to the international scientific community.

**Case Example – Pro-active Marketing^14^**

The Tumour Bank at the Children’s Hospital Westmead (TB-CHW) is a single site BB with a research focus on rare paediatric malignancies.^14^ They have been pro-active when forming collaborations. The TB-CHW actively sought out international, leading world experts whose research and results would most likely lead to a greater understanding of childhood cancer through the addition of TB-CHW samples. This novel approach has facilitated 84 research projects around the world, resulting in over 40 genomic-based research publications.^14^ Interestingly, 76% of research publications were from collaborations between international researchers, underscoring the importance of a single site RD registry with a BB to the international research community.^14^

**Case Example – Recruitment^34^**

The Canadian Neuromuscular Disease Registry (CNDR) is a national registry established in 2011.^34^ The CNDR was established to bridge the gaps of knowledge for rare, neuromuscular diseases in Canada. The registry utilised a novel recruitment approach to “cast the net” as far as possible and reach patients affected by neuromuscular disease. Blended recruitment, the term given to this model, offers several methods for recruiting patients. In addition to the traditional approach of clinicians enrolling patients during routine visits to clinic, the CNDR also offers direct self-registration through the registries main office, with the option of patients registering themselves through the CNDR public website, connecting interested patients with CNDR staff at head office. Blended recruitment offers a novel approach to recruitment, enabling both clinicians and patients to recruit through several means. As a result, the CNDR recruited 253 Duchenne and Becker Muscular Dystrophy patients, 161 myotonic dystrophy patients, and 71 ALS patients. Enrolment extended to 12 provinces and territories, confirming the feasibility and efficacy of blended recruitment for RD registries.

**Case Example – Challenges^19^**

Whilst the advent of next-generation sequencing (NGS) provides unparalleled opportunities for RD research, the TNGB found limitations when implementing this technology due to legal and ethical concerns. ^19^ It was noted their current informed consent was restricted to that patient’s particular disease, and lacked the necessary broad consent to implement NGS. Moreover, governing the sheer volume of information generated by NGS required additional considerations. This was especially so when managing “incidental findings”. In response, the TNGB collaborated with experts in order to accelerate national regulations for BB.
